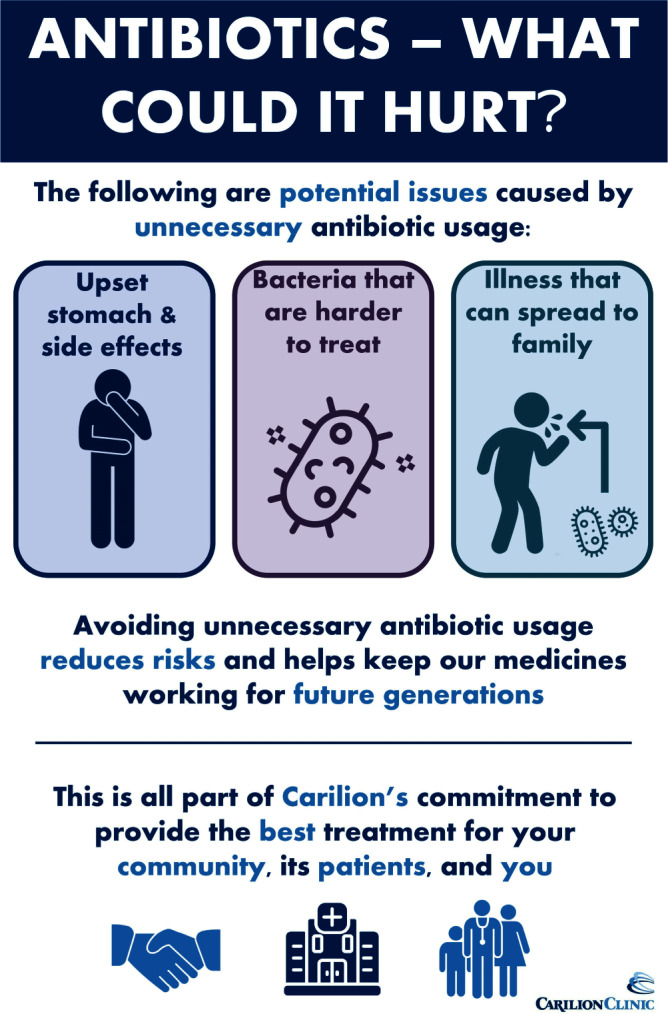# Patient and Community Perspectives on Antibiotics and Antimicrobial Resistance: Fertile Grounds for Antimicrobial Stewardship

**DOI:** 10.1017/ash.2024.152

**Published:** 2024-09-16

**Authors:** Anthony Baffoe-Bonnie, Mandy Swann, Carter Gottschalk, Brent Brewer, Nathan Everson

**Affiliations:** Virginia Tech Carilion School of Medicine; Carilion Clinic

## Abstract

**Background:** Antimicrobial resistance is a serious public health threat. Overuse of antibiotics leads to the development and spread of antibiotic resistant pathogens. Antibiotics are also responsible for a high percentage of emergency department visits for adverse drug events. Despite this, ambulatory and urgent care providers often cite patient expectations as a reason for inappropriate antibiotic prescribing practices. We investigated patient and community understanding of antibiotics and antimicrobial resistance to inform how they can be engaged as partners in combating antimicrobial resistance in our southwestern Virginia community. **Methods:** From July to September 2023, we conducted an online survey of patients and community members within the footprint of a large healthcare system in Southwest Virginia. Electronic medical records were used to randomly select and directly email the survey link to a representative sample of ambulatory patients who met criteria. Respondents were also recruited through the health system’s social media channels and through posters with quick response (QR) codes in outpatient offices. The survey used Likert scales and multiple-choice questions to understand experiences with and perceptions about antibiotics and antimicrobial resistance. We conducted a descriptive analysis of survey responses. **Results:** In total, 2,021 individuals completed the survey. Nearly 16% of respondents agreed with the statement “antibiotics can kill viruses” and almost 12% more were unsure. Thirty percent of respondents either agreed with or were unsure about the statement “antibiotics work on most coughs and colds”. When asked more directly about antimicrobial resistance, almost a quarter (25%) of respondents agreed with or were unsure about the statement “there is no connection between taking antibiotics and the development of resistant bacteria”. Responding to questions about possible negative effects of antibiotics, over 9% disagreed with the statement “antibiotics can kill the 'good' bacteria that normally live on the skin and in the gut” and another 19% were unsure. Similarly, over 20% disagreed with or were unsure about the statement “bacteria that do not respond to antibiotics could infect me or my family”. Reflecting on their own providers, nearly 83% of respondents trusted their doctor’s or nurse’s advice about antibiotic necessity. **Conclusions:** There are opportunities for patient and community engagement around antibiotic effectiveness for common viral illnesses and about the negative effects of overuse of antibiotics. Our data suggests most patients trust their providers as it relates to antibiotic prescribing and may be receptive to discussions and strategies that promote antimicrobial stewardship.

**Figure:**